# A role for nematocytes in the cellular immune response of the Drosophilid *Zaprionus indianus*

**DOI:** 10.1017/S0031182013001431

**Published:** 2014-01-28

**Authors:** Balint Z. Kacsoh, Julianna Bozler, Todd A. Schlenke

**Affiliations:** Biology Department, Emory University, 1510 Clifton Road NE, Atlanta, GA 30322, USA

**Keywords:** Zaprionus, encapsulation, haemocyte, nematocyte, endoparasitoid wasp

## Abstract

The melanotic encapsulation response mounted by *Drosophila melanogaster* against macroparasites, which is based on haemocyte binding to foreign objects, is poorly characterized relative to its humoral immune response against microbes, and appears to be variable across insect lineages. The genus Zaprionus is a diverse clade of flies embedded within the genus *Drosophila*. Here we characterize the immune response of *Zaprionus indianus* against endoparasitoid wasp eggs, which elicit the melanotic encapsulation response in *D. melanogaster*. We find that *Z. indianus* is highly resistant to diverse wasp species. Although *Z. indianus* mounts the canonical melanotic encapsulation response against some wasps, it can also potentially fight off wasp infection using two other mechanisms: encapsulation without melanization and a non-cellular form of wasp killing. *Zaprionus indianus* produces a large number of haemocytes including nematocytes, which are large fusiform haemocytes absent in *D. melanogaster*, but which we found in several other species in the subgenus Drosophila. Several lines of evidence suggest these nematocytes are involved in anti-wasp immunity in *Z. indianus* and in particular in the encapsulation of wasp eggs. Altogether, our data show that the canonical anti-wasp immune response and haemocyte make-up of the model organism *D. melanogaster* vary across the genus Drosophila.

## INTRODUCTION

Cellular encapsulation of pathogens by haemocytes (blood cells) is common to diverse invertebrate hosts (Salt, 1963, 1970; Ratner and Vinson, 1983; Gotz, 1986; Lackie, 1988; Gillespie *et al.* 1997), is important for pathogen resistance in insect vectors of human disease (Richman and Kafatos, 1996) and is functionally similar to granuloma formation in vertebrates (Adams, 1976). In this immune response, pathogens in the haemolymph (blood) are recognized as foreign, which activates haemocytes to migrate towards, adhere to, and consolidate around the pathogen, forming a multi-cellular multi-layered capsule. The capsule is thought to form a physical barrier to pathogen escape, but pathogens may also be actively killed by free radicals released by the host inside the capsule (Nappi *et al.* 1995, 2000; Nappi and Vass, 1998; Kraaijeveld *et al.* 2011). In *Drosophila melanogaster* and some other organisms, encapsulation is accompanied by melanization, the deposition of melanin around the pathogen inside the capsule, which is thought to strengthen the physical barrier to larval egg hatching (Gillespie *et al.* 1997; Carton *et al.* 2008; Nappi *et al.* 2009). Cellular encapsulation usually occurs in response to pathogens that are too large or too numerous to be phagocytosed by individual haemocytes.

*Drosophila melanogaster* haemocytes have been divided into four classes: (1) plasmatocytes are small cells that comprise ~ 95% of all haemocytes in un-induced flies, act as sentinels of infection, and are responsible for phagocytosis in addition to serving as the initial cell layers of developing capsules; (2) crystal cells comprise the remaining 5% of standing haemocytes and carry precursors for generating melanin, which is important in wound healing and is deposited around encapsulated objects; (3) podocytes are medium-sized cells induced after infection that contain numerous filopodia, or cytoskeletal extensions, and are considered an intermediate form between plasmatocytes and lamellocytes; (4) lamellocytes are large flattened cells that differentiate after infection from plasmatocytes and from lymph gland pro-haemocytes, and they are responsible for forming the outside cellular layers of developing capsules (Rizki, 1957; Rizki and Rizki, 1980; Carton *et al.* 1986; Russo *et al.* 1996; Williams, 2007; Honti *et al.* 2009; Markus *et al.* 2009; Stofanko *et al.* 2010). Thus, plasmatocytes and lamellocytes are the main cellular components of *D. melanogaster* capsules. However, because haemocyte morphology is highly variable across invertebrate lineages, organization of a general haemocyte classification scheme has been difficult and many different named haemocyte classes participate in the encapsulation responses of different host species (Wigglesworth, 1959; Jones, 1962; Rizki, 1962; Lackie, 1988; Gupta, 1994; Lavine and Strand, 2002).

In order to study cellular encapsulation and other mechanisms of immunity against macroparasites, a suitable parasite must be chosen. Endoparasitoid wasps are ubiquitous, act as keystone species in natural ecosystems, are used in biocontrol of insect pests, and often induce a cellular encapsulation response when they lay their eggs in the haemocoels of their hosts (LaSalle and Gauld, 1993). These wasps are a serious threat to juvenile Drosophila as upwards of 50% of fly larvae are found to be infected in natural populations (Driessen *et al.* 1990; Janssen *et al.* 1988; Fleury *et al.* 2004). Four wasp families are known to infect Drosophila in nature: members of the Braconidae and Figitidae infect Drosophila larvae, while members of the Diapriidae and Pteromalidae infect Drosophila pupae (Carton *et al.* 1986). Wasps inject venom into flies, along with their eggs, in order to suppress the fly encapsulation response, but venoms from the endoparasitoids of Drosophila are only partially characterized. Virus-like particles with immuno-suppressive properties have been found in the venom glands of some Figitids, but presumably the majority of wasp virulence proteins are made independently in the venom glands and ovaries (Carton *et al*. 1986, 2008; Colinet *et al*. 2013; Goecks *et al*. 2013; Mortimer *et al*. 2013). If the wasp eggs successfully hatch, the wasp larvae grow within the fly larvae and pupae for several days before ultimately consuming the fly pupae from the inside out, eclosing from the host pupal cases. The success of the cellular encapsulation response against wasps across the *D. melanogaster* subgroup has been found to be strongly correlated with constitutive and induced haemocyte loads (Eslin and Prevost, 1998; Kraaijeveld *et al.* 2001; Sorrentino *et al.* 2004; Moreau *et al.* 2005; Kacsoh and Schlenke, 2012). However, it is unknown whether this relationship extends across the entire genus Drosophila or even whether more distant Drosophila species utilize the same melanotic encapsulation mechanism for killing wasp endoparasitoids.

**Fig. 1:**
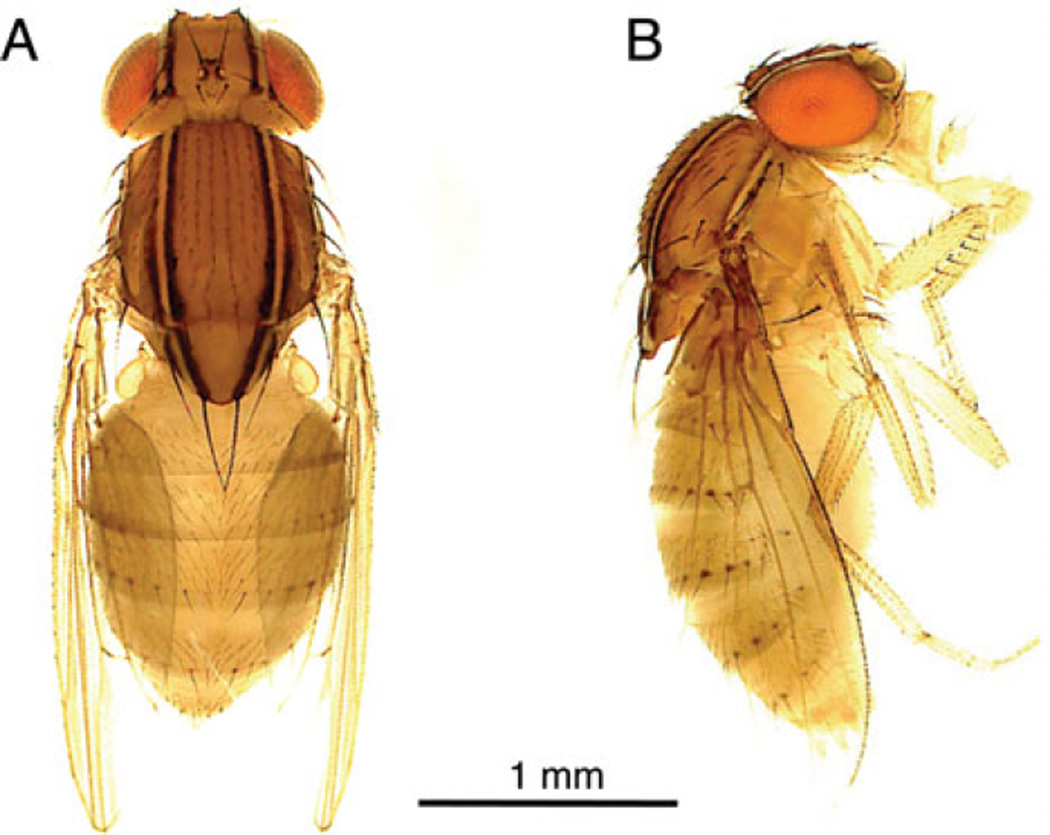
*Zaprionus indianus*. Female dorsal view (A) and lateral view (B).

**Fig. 2:**
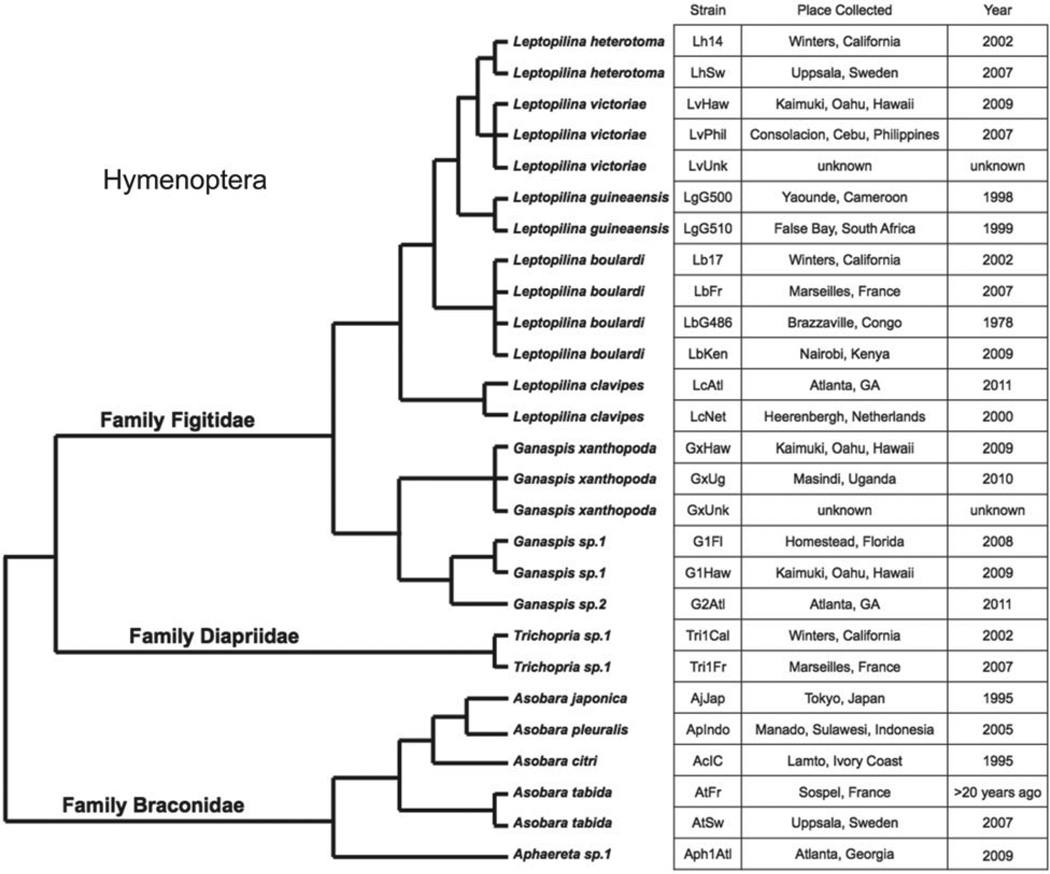
Phylogenetic relationships and provenance of parasitic wasps used in this study. Tree topology and branch lengths are approximated from studies of Hymenopteran family relationships (Dowton, 2001), Figitid relationships (Schilthuizen *et al.* 1998; Allemand *et al.* 2002), and Braconid relationships (Seyahooei *et al.* 2011).

The genus Zaprionus comprises more than 50 described species (Chassagnard and Tsacas, 1993) and is known to be phylogenetically embedded within the genus Drosophila, although its affinity to particular Drosophila subgenera is still unclear (Markow and O’Grady, 2006). Zaprionus is distinctive in that all species possess distinct (usually longitudinal) light-coloured stripes (Fig. 1). The species *Zaprionus indianus*, in particular, is native to Africa, the Middle East and southern Eurasia, but has recently gained attention because of its spread to North and South America where it causes severe economic damage as a pest species of figs (Santos *et al.* 2003; Stein *et al.* 2003; van der Linde *et al.* 2006). Parasitic wasps have been successfully used as biocontrol agents against a wide range of agricultural pests (Huffaker, 1971; Debach, 1974; Clausen, 1978; Greathead, 1986; LaSalle and Gauld, 1993), but have not as yet been used against Drosophilids. Given *Z. indianus* is part of a unique Drosophilid subgenus and is a growing agricultural threat, we decided to test its mechanism(s) of resistance against a diverse panel of Drosophila parasitic wasps (Fig. 2). This panel includes representatives from three of the four wasp families known to infect Drosophila as well as multiple strains from a total of 15 wasp species. As of yet, very little is known about the interaction between *Z. indianus* and parasitic wasps in nature, other than it can be infected by the Figitid *Dicerataspis grenadensis* (Guimaraes and Zucchi, 2004). Other Zaprionus species have been found to serve as suitable hosts for the Figitid *Leptopilina heterotoma* (Jenni, 1951).

## MATERIALS AND METHODS

### Insects

A large population of *Z. indianus* were collected from fruit traps in Atlanta, GA in the summer of 2011 by T.A.S. From this population, three independent inbred lines were generated through 10 generations of sib-sib inbreeding. Assaying multiple Z. *indianus* strains ensures that the conclusions drawn are representative of the species and not an outlier strain. The flies were maintained in the lab on standard *D. melanogaster* media and kept in a 25 °C incubator with 12–12 day-night cycles. Drosophila species strains assayed for nematocytes were ordered from the Drosophila Species Stock Center or were collected by us (*Drosophila suzukii*) or by others (*Drosophila putrida, Scaptodrosophila lebanonensis*).

A total of 27 Drosophila parasitoid wasp strains were used for infection trials on *Z. indianus* (Fig. 2). Strains LgG500 and LgG510 were provided by R. Allemand, strain LbG486 was provided by D. Hultmark, strains LcNet, AjJap, ApIndo and AcIC were provided by J. van Alphen, strain GxUg was provided by J. Pool and strain AtFr was provided by B. Wertheim. All other strains were collected by the Schlenke lab. These wasp strains represent: (i) at least 14 species, (ii) representatives of three of the four *Hymenopteran* families known to infect *Drosophila*, (iii) larval and pupal endoparasitoids, and (iv) a worldwide range of collection localities. Morphology and cytochrome oxidase I (COI) sequences from the two *Trichopria* sp. strains suggested they are representatives of the same species, perhaps *Trichopria drosophila*. Furthermore, morphology and COI sequences from the two *Ganaspis* sp. 1 strains suggest they are representatives of a single undescribed species.

To grow wasps, adult flies were allowed to lay eggs in standard *Drosophila* medium for several days before they were replaced by adult wasps, which then attacked the developing fly larvae or pupae. Wasp vials were supplemented with approximately 500 µL of a 50% honey/water solution applied to the inside of the cotton vial plugs. All wasp species were maintained in the laboratory on *D. melanogaster* strain Canton S, with the exception of *Leptopilina victoriae*, which was maintained on *Drosophila ananassae*, and *Asobara tabida, Aphaereta* sp., *Ganaspis* sp. 2, *Leptopilina clavipes* and *Leptopilina guineaensis* strain LgSA, which were maintained on *Drosophila virilis. COI* sequences for all wasp strains as well as ITS2 sequences for Figitid wasps have been deposited in GenBank under accession numbers DQ218153– DQ218154 and JQ808406–JQ808451. Wasp strains are available upon request.

### Resistance trials

Adult female *Z. indianus* were allowed to lay eggs into 60 mm Petri dishes filled with a molasses medium supplemented with yeast paste. After 96 h, adult flies were removed and second instar fly larvae were collected to perform the wasp infections. For wasp attacks on fly larvae, 50 fly larvae were moved onto a 35 mm Petri dish filled with 1 mL of standard Drosophila medium. Three female wasps were then placed onto the dish and allowed to attack for 72 h. After the attack period, 10 of the 50 larvae were dissected to assay the number of wasp eggs laid per larva and the proportion of fly larvae bearing at least one encapsulated wasp egg. 30 of the remaining 40 larvae were then moved into vials containing Drosophila medium and allowed to complete development. The remaining 10 larvae served as extras in case any accidental mortality occurred in moving larvae between dishes/vials. For the pupal endoparasitoids, 40 third instar larvae were placed into a vial containing Drosophila medium. Three female wasps were then placed into the vial and allowed to attack for 72 h, after which the fly development was allowed to proceed to completion.

Infection conditions were designed to optimize wasp success and control uninfected flies were reared under identical conditions and showed 100% survival (data not shown). The total number of flies and wasps eclosed from the treatments were determined 17 and 32 days following infection, respectively, times by which all viable flies and wasps should have eclosed. The fly–wasp interaction yielded three possible outcomes: (1) fly eclosion following a successful immune response; (2) wasp eclosion following successful immune suppression; or (3) death of both the fly and the wasp. Three infection trial replicates were run for each fly–wasp interaction.

### Haemocyte counts

*Zaprionus indianus* larval haemocyte counts were made following a sterile needle pierce or following infection by four wasp species with varying virulence patterns (two virulent, two avirulent). Piercing larvae with a needle acts as a control for the cuticle puncture associated with wasp attack and is known to cause induction of lamellocyte production in *D. melanogaster* (Markus *et al.* 2005). For the piercing experiment, 15 size-matched 96 h old *Z. indianus* larvae were rinsed in 1× PBS and dried on Kimwipes. They were then immobilized on double-sided tape and their posterior cuticles were pierced by a flame-sterilized 0·1 mm diameter stainless steel dissecting pin. Care was taken to avoid piercing internal organs. The larvae were then removed with a wet paintbrush and placed into a moist chamber for 1 h to allow for recovery before being moved to 35 mm diameter Petri dishes filled with 1 mL Drosophila medium. Haemocytes were counted 24 h following treatment. To measure constitutive haemocyte levels, larvae were treated identically but without the pierce. All treatments were performed in five replicates. For haemocyte counts following wasp infection, fifteen 72 h old second instar larvae were exposed to three female wasps for 24 h. Haemocytes were counted 24 h after the end of wasp exposure in three replicates per treatment.

In order to count haemocytes, 5 third instar larvae were removed from each replicate dish, rinsed in Drosophila Ringer’s solution, dried on a Kimwipe, and bled together into 20 µL of 1× PBS solution containing 0·01% phenylthiourea to prevent melanization (Lerner and Fitzpatrick, 1950). This buffer-haemolymph mixture was then pipetted into a disposable haemocytometer (Incyto C-Chip DHC-N01) and allowed to sit for 30 min to allow the haemocytes to settle. Haemocytes from each sample were counted from sixteen 0·25×0·25×0·1 mm squares. The number of haemocytes in the whole sample is expected to be 200 times the number counted, and the number of haemocytes per fly larva is expected to be 40 times the number counted. Haemocyte counts may be underestimates for a variety of reasons. For example, in *D. melanogaster* a large proportion of plasmatocytes are sessile and may not be extracted with the haemolymph (Lanot *et al.* 2001; Markus *et al.* 2009). There also may be an interaction between haemocyte counts and wound/infection status, e.g. lamellocytes are removed from circulation when they adhere to wasp eggs.

Crystal cells are difficult to count and image because they rapidly lose their crystals following dissection. However, *D. melanogaster* crystal cells have been shown to self-melanize when larvae are incubated at 60 °C for 10 min (Rizki and Rizki, 1980; Williams *et al.* 2005). We optimized the incubation protocol for *Z. indianus*, incubating 5 larvae from each piercing/constitutive treatment at 63 °C for 45 min before counting blackened crystal cells on the dorsal surface of intact larval cuticles.

### Imaging

Fly larvae from the haemocyte count experiments were used for haemocyte imaging using one of two protocols. First, the buffer-haemolymph mixture described above was applied to a glass slide with coverslip and imaged at 1000× under phase contrast microscopy to yield haemocyte images corresponding in structure to the haemocyte types observed in the haemocytometer counting experiments. Second, to gain a more detailed view of haemocyte internal structure, whole larvae were submersed in a drop of microscope immersion oil and dissected open to release their haemolymph underneath the oil, which was then covered by a coverslip (Rizki, 1953). Pressure from the oil flattens the haemocytes against the slide surface, making the cells appear larger and enhancing cell structural details.

For cytoskeletal staining of haemocytes, 50 *Z. indianus* larvae were attacked by the wasp strain LvPhil for 24 h. Following wasp attack, individual larvae were bled into 20 µL of Drosophila Ringer’s solution and haemocytes were allowed to adhere to a glass slide for 30 min at room temperature. The cells were then fixed in 4% paraformaldehyde for 15 min and washed once with 1× PBS followed by a 10 min incubation in 0·01% Triton X-PBS. Cells were washed once more with 1× PBS and then stained with 0·2 µg µL^−1^ TRITC-phalloidin in 3% BSA-PBS for 30 min. Counter staining was performed with 2 µg mL^−1^ DAPI-PBS for 5 min, prior to a final wash with 1× PBS.

To image encapsulated wasp eggs, wasp eggs were dissected out of fly larvae onto a glass slide into 20 µL of Ringer’s solution and observed under phase contrast microscopy. It was previously shown that insect haemocytes involved in encapsulation will disassociate from the capsule over time when incubated in a modified Ringer’s solution containing 1% EDTA, which disrupts calcium-mediated cell adhesion (Reik, 1968). We used this method to identify the *Z. indianus* haemocyte classes involved in the encapsulation process.

### Statistical analysis

**Fig. 3:**
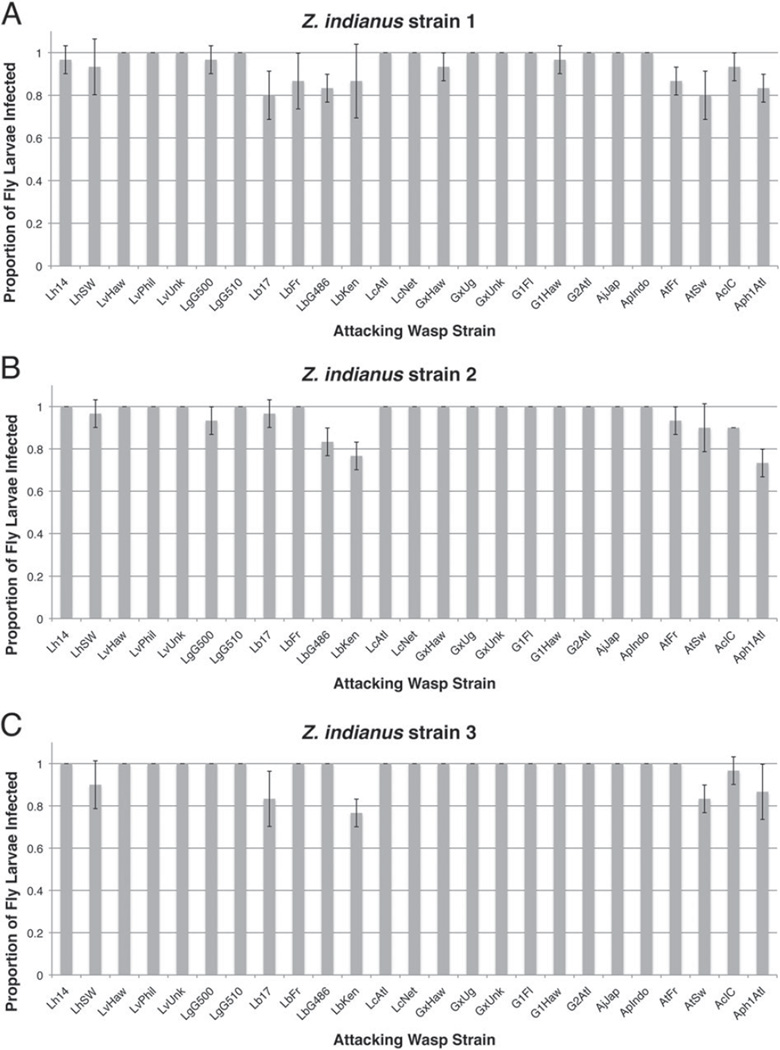
*Zaprionus indianus* infection rates across wasp strains. The proportion of larvae infected by at least one wasp egg was measured for each fly–wasp interaction using *Z. indianus* strains 1 (A), 2 (B), and 3 (C). The mean (±) 95% confidence intervals are shown for three replicates of each fly–wasp pair. Infection rate was not measured for pupal endoparasitoids.

**Fig. 4:**
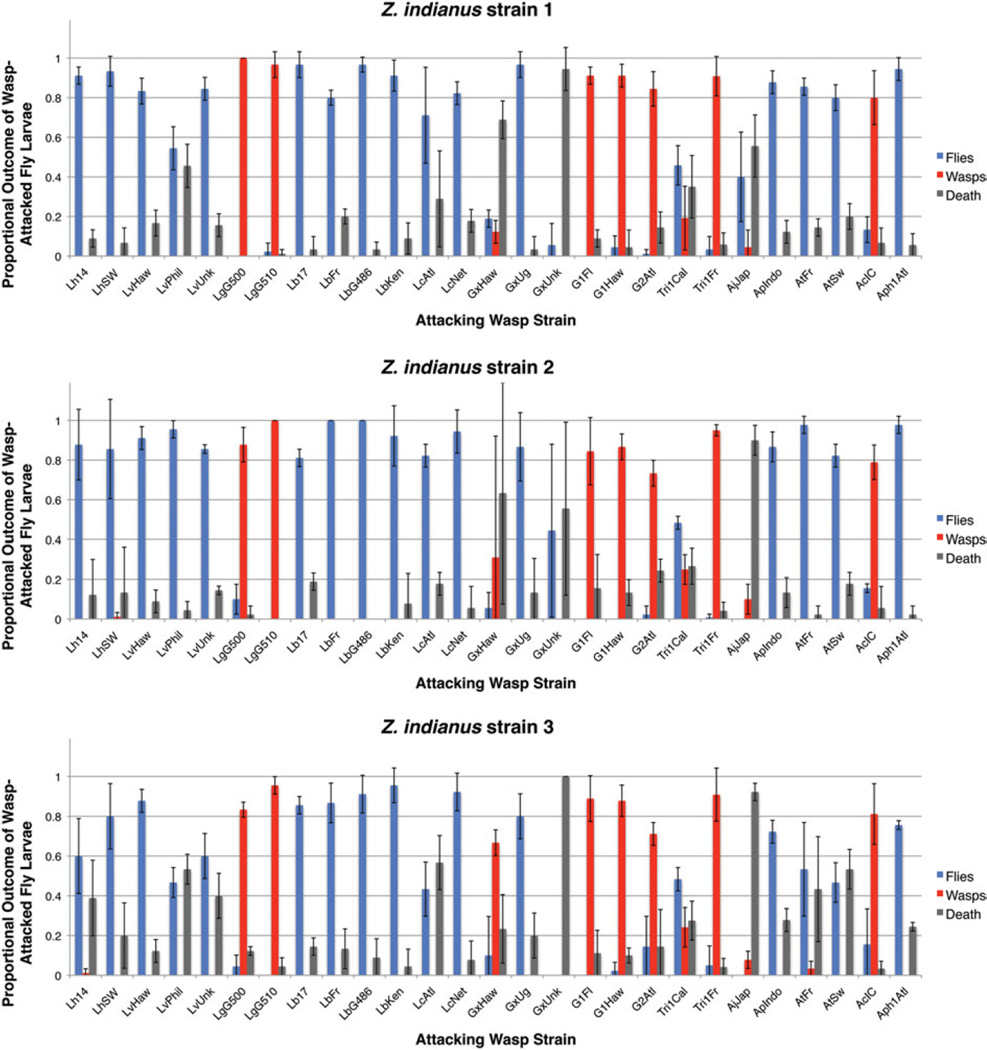
Outcomes for wasp-infected *Z. indianus*. The proportion of flies and wasps that eclosed from each fly–wasp interaction, as well as the proportion of wasp-infected flies that died, is shown for *Z. indianus* strains 1 (A), 2 (B), and 3 (C). The mean (±) 95% confidence intervals are shown for three replicates of each fly–wasp pair.

All statistical analyses were run in R version 2.10.1. For analyses of proportion data, e.g. proportion of fly larvae infected (Fig. 3), proportion of flies and wasps eclosed (Fig. 4), and proportion of larvae that have encapsulated wasp eggs (Fig. 6), comparisons between fly strains and between wasp strains were made using general linear models with quasibinomial errors. For analyses of haemocyte count data (Figs 11 and 13), general linear models with quasi-Poisson errors were used.

## RESULTS

### Fly resistance

We exposed each of three *Z. indianus* strains to infection by 27 parasitoid wasp strains. Since we did not observe the wasps during the exposure period, we first assayed the number of eggs laid by the 25 larval parasitoid wasp strains in exposed fly larvae to ensure they had been infected at a high frequency. The average proportion of fly larvae found to be infected by at least one wasp egg was, as expected given the ratio of flies to wasps and the long infection period, very high for all fly–wasp combinations (Fig. 3), and no significant differences were found between *Z. indianus* strains (Tukey contrasts, all *P*>0·188). Although there were significant differences between wasp strains in infection frequency (wasp strains LbKen and Aph1Atl were the lowest, averaging ~ 80%), the overall high infection proportions indicate that almost all adult fly eclosion observed must be due to flies mounting successful immune responses rather than avoiding wasp parasitism.

The outcomes of any fly–wasp infection are fly eclosion due to a successful immune response, wasp eclosion due to a successful virulence strategy, or a host–parasite incompatibility leading to death of both fly and wasp. We found remarkably consistent infection outcomes across the three *Z. indianus* strains (Fig. 4). There was no significant difference between fly strains in any infection outcome (fly eclosion, wasp eclosion, death) after infection by the panel of wasps (Tukey contrasts, all *P*>0·137), with the exception of a marginally significantly increased amount of death in *Z. indianus* strain 3 compared with strain 2 (Tukey contrast, *P* = 0·022). There were, however, consistent significant differences in infection outcomes across wasp strains. The wasp species *L. guineaensis, Ganaspis* sp. 1, *Ganaspis* sp. 2, *Trichopria* sp. 1 and *Asobara citri* were all highly successful infectors of *Z. indianus*, the wasps *Ganaspis xanthopoda* and *Asobara japonica* induced a high proportion of death in *Z. indianus* hosts, and *Z. indianus* was relatively resistant against all other wasp species. Given *L. guineaensis* and *A. citri* are thought to be native to Africa and to overlap with the ancestral *Z. indianus* home range, it is tempting to speculate that they have adapted to specialize on *Z. indianus* hosts. While possible, the other successful *Z. indianus* infectors are not known to have overlapping ranges with *Z. indianus* (Carton *et al.* 1986). Furthermore, *L. guineaensis* and *A. citri* are successful infectors of other Drosophilid species as well, and other wasps with African ranges such as *Leptopilina boulardi* and *G. xanthopoda* have poor success on *Z. indianus* hosts. Thus, there seems to be little if any correspondence between wasp species distributions and infection success against *Z. indianus*. Although field studies will be required to uncover the natural wasp endoparasitoids of *Z. indianus*, the five successful infector species identified here are candidates for use in *Z. indianus* biocontrol in agricultural settings.

### Encapsulation and other mechanisms of wasp killing

**Fig. 5:**
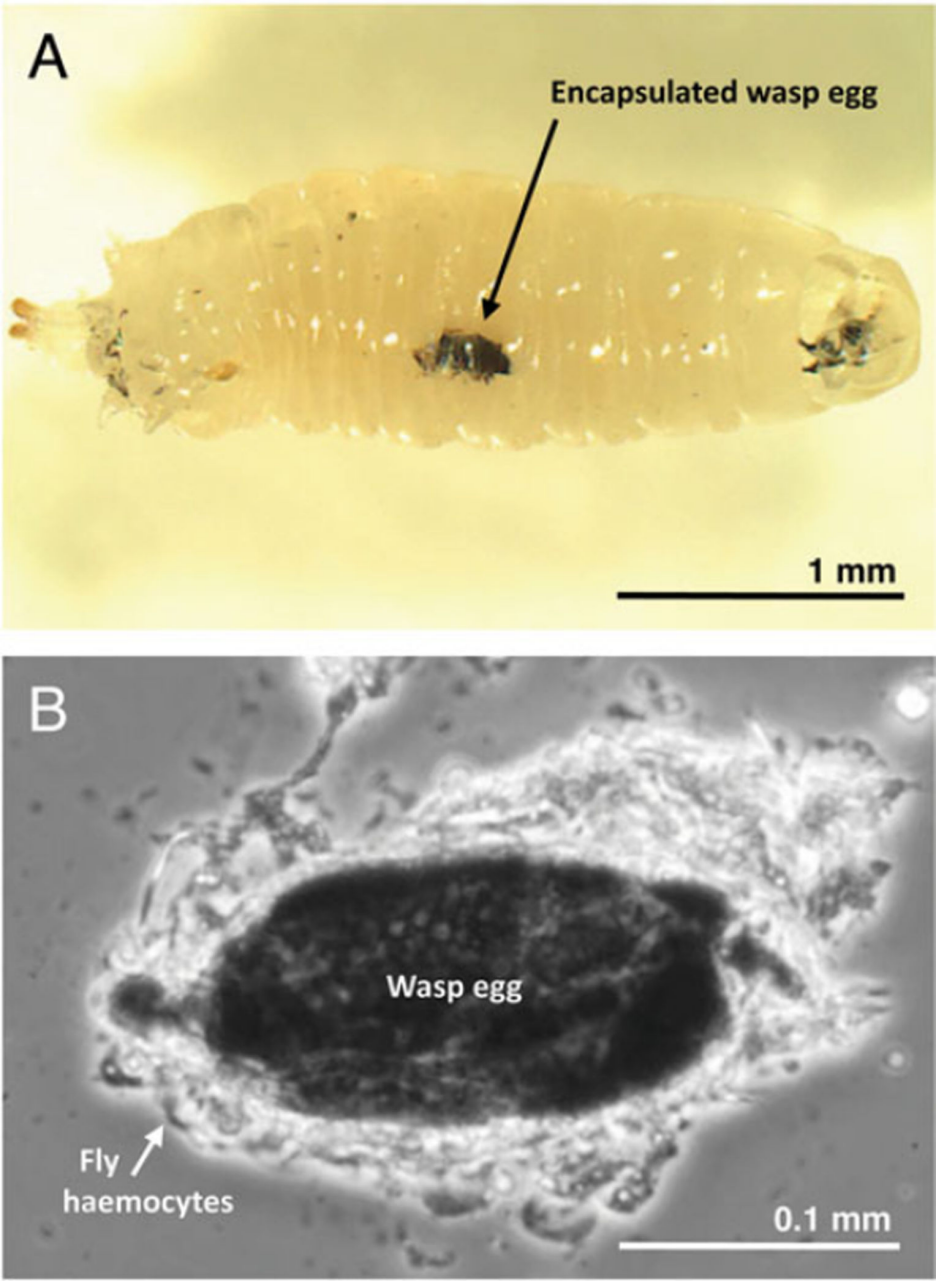
Melanotic encapsulation of wasp eggs. (A) A *Z. indianus* larva with an encapsulated egg from wasp strain LvPhil; (B) an encapsulated wasp egg dissected from a *Z. indianus* larva showing multiple layers of haemocytes making the capsule.

**Fig. 6:**
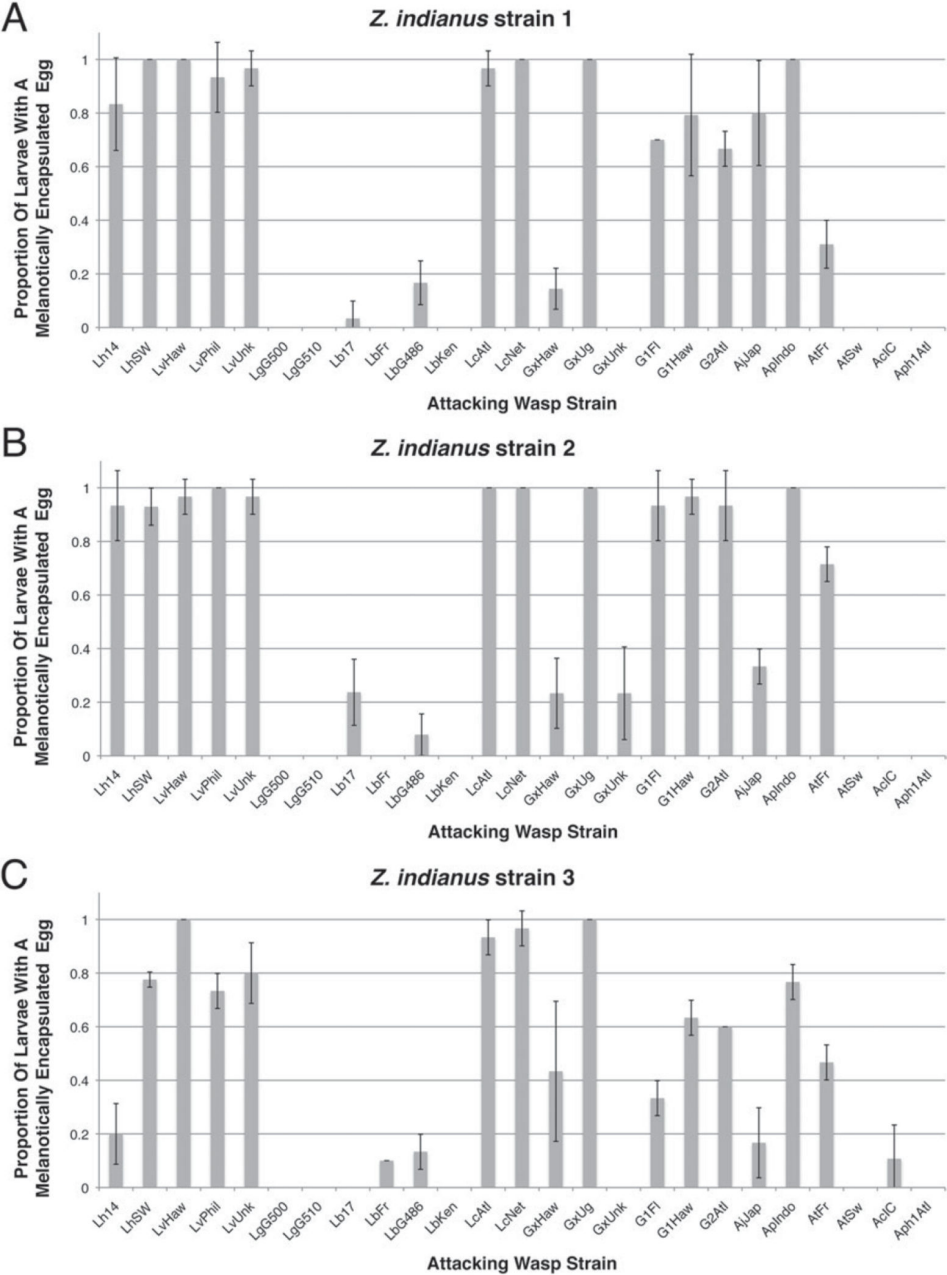
Melanotic encapsulation success against a panel of wasp larval endoparasitoids. The proportion of infected fly larvae that melanotically encapsulated at least one wasp egg is shown for *Z. indianus* strains 1 (A), 2 (B), and 3 (C). The mean (±) 95% confidence intervals are shown for three replicates of each fly–wasp pair.

We found that *Z. indianus* can use the canonical melanotic encapsulation response described from *D. melanogaster* to kill the eggs of some wasp species. For example, blackened wasp eggs were readily observed through the cuticle of intact *Z. indianus* larvae infected by *L. victoriae* (Fig. 5A), and there were distinct consolidated cellular capsules around the outside of melanized eggs dissected from these fly larvae (Fig. 5B). *Z. indianus* was able to melanotically encapsulate eggs from a total of 11 of 13 larval parasitoid wasp species, often in high proportions, and there was no significant difference in encapsulation success between *Z. indianus* strains (Fig. 6, Tukey contrasts, all *P*>0·127). However, there were consistent significant differences in encapsulation success across wasp strains. *Zaprionus indianus* never melanotically encapsulated eggs from the wasps *L. guineaensis* or *Aphaereta* sp., and rarely did so for the wasps *L. boulardi* and *A. citri*. In some cases there was variation within wasp species in the propensity to be melanotically encapsulated by *Z. indianus*. For example, wasp strain GxUg had eggs encapsulated in almost every fly larva, while strains GxHaw and GxUnk had eggs melanotically encapsulated very rarely. Likewise, wasp strain AtFr had eggs encapsulated at appreciable frequency, but eggs from strain AtSw were never melanotically encapsulated. These differences suggest that there is substantial genetic variation in the ability to suppress *Z. indianus* immunity within wasp species.

**Fig. 7:**
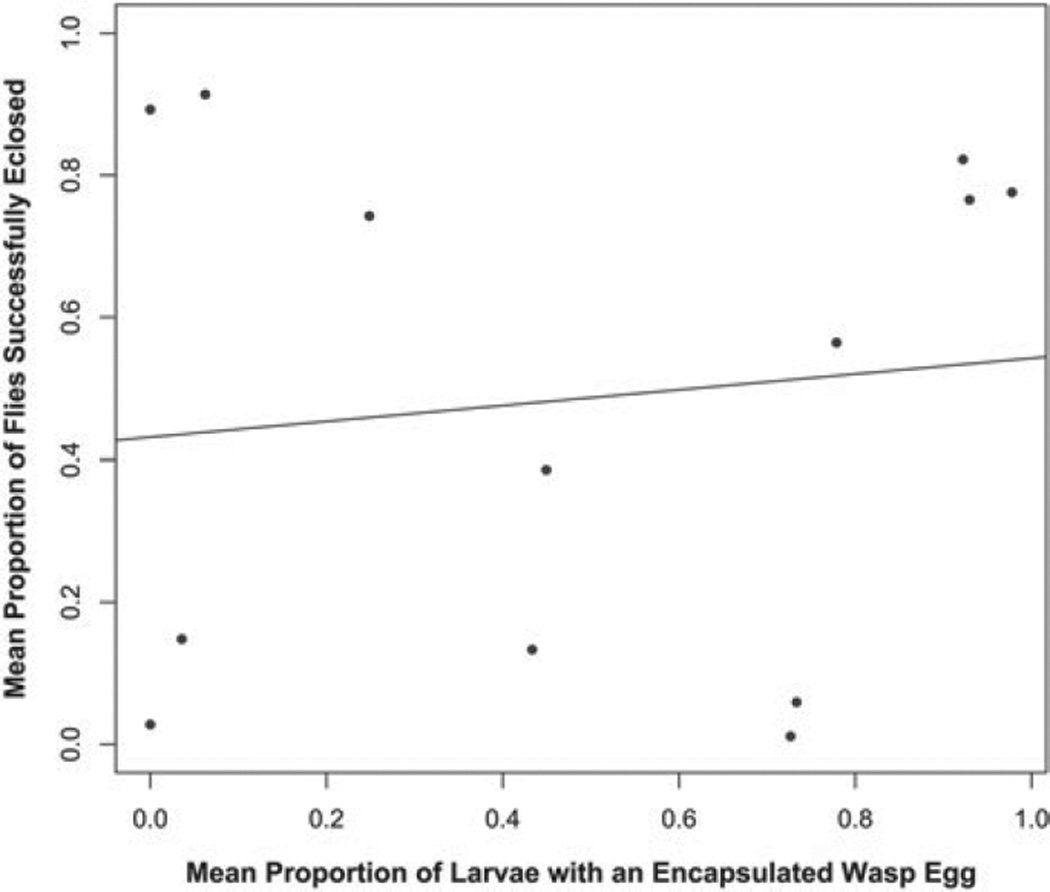
Lack of correlation between fly encapsulation success and eclosion success. The mean proportion of infected *Z. indianus* larvae that encapsulated at least one wasp egg was compared to the mean eclosion success of *Z. indianus* against each wasp, using combined data from the three *Z. indianus* strains. Data from wasp species strains were averaged into single species values, and pupal endoparasitoids were not considered.

If melanotic encapsulation is the sole method by which *Z. indianus* kills wasp eggs, we should expect a strong correlation between *Z. indianus* encapsulation success and eclosion success across the panel of wasps. However, no such correlation exists (*r*^2^ = 0·011, *P* = 0·733) (Fig. 7). A comparison of the eclosion patterns (Fig. 4) with the encapsulation patterns (Fig. 6) reveals three potential explanations: First, some wasps appear to be frequently encapsulated by *Z. indianus* but nevertheless eclose from the flies in high numbers (e.g. *Ganaspis* sp. 1, *Ganaspis* sp. 2). This is due in large part to cases in which multiple wasp eggs were laid per fly but the flies only melanotically encapsulated a portion of them, leaving at least one healthy juvenile wasp to develop and eclose. Second, for some wasps that *Z. indianus* was regularly able to melanotically encapsulate, the flies later died and no flies or wasps eclosed, suggesting the flies mounted an over-reactive immune response (e.g. *L. victoriae, A. japonica*). Third, *Z. indianus* successfully eclosed after infection by some wasp species despite mounting no melanotic encapsulation response (e.g. *L. boulardi, A. tabida, Aphaereta* sp.), suggesting this fly might have other means of killing wasp eggs or larvae.

**Fig. 8:**
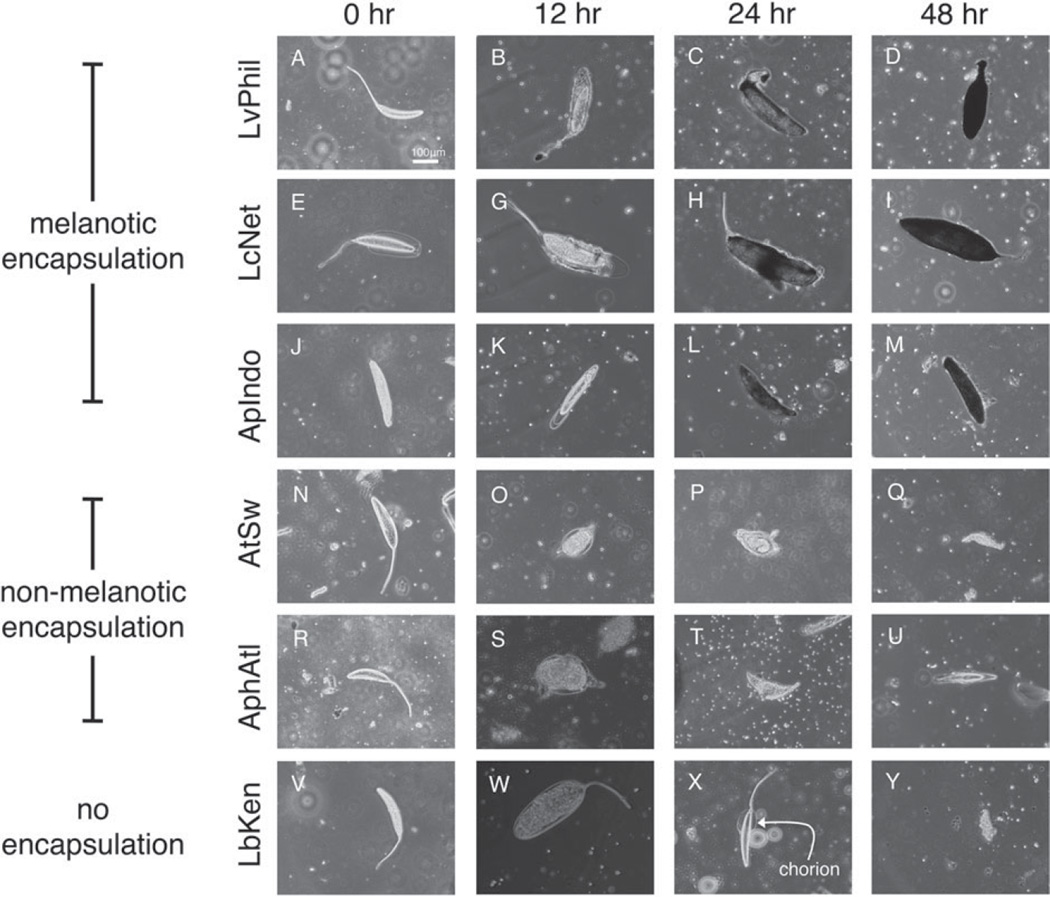
Three wasp egg-killing strategies used by *Z. indianus*. Time course images were taken of wasp eggs dissected from infected fly larvae. *Zaprionus indianus* killed eggs of some wasp species using melanotic encapsulation (A–M), eggs of other wasp species using non-melanotic encapsulation (N–U), and appear to kill *L. boulardi* eggs using a non-cellular mechanism of dissolving away the egg chorions (V–Y).

To elucidate the alternative wasp killing mechanisms used by *Z. indianus*, we dissected wasp eggs from flies at different stages of development for six representative wasp strains (Fig. 8). As expected, wasp strains LvPhil, LcNet and ApIndo showed a similar pattern of melanotic encapsulation, where individual fly haemocytes were observed bound to the outside of the wasp eggs starting at 12 h post-infection, with melanization of the eggs starting at 24 h post-infection. This is similar to the progression of melanotic encapsulation in *D. melanogaster* (Russo *et al.* 1996, 2001; Lanot *et al.* 2001). Eggs from wasp strains AtSw and AphAtl also had fly haemocytes bound to them starting at 12 h post-infection. This encapsulation seemed to result in a dramatic change in the shape of the wasp eggs from elongate to circular, and the eggs died without ever becoming melanized. Finally, eggs from wasp strain LbKen were never encapsulated but seemed to lose their chorion layer at 24 h post-infection (Fig. 8X), resulting in egg cell lysis and wasp death. This could be the result of an undefined humoral immune response mounted by *Z. indianus* or a lack of appropriate developmental resources for LbKen in *Z. indianus* hosts. Thus, *Z. indianus* uses two to three distinct wasp egg-killing strategies: melanotic encapsulation, non-melanotic encapsulation and a possible humoral response.

### Haemocyte classification and counts

**Fig. 9:**
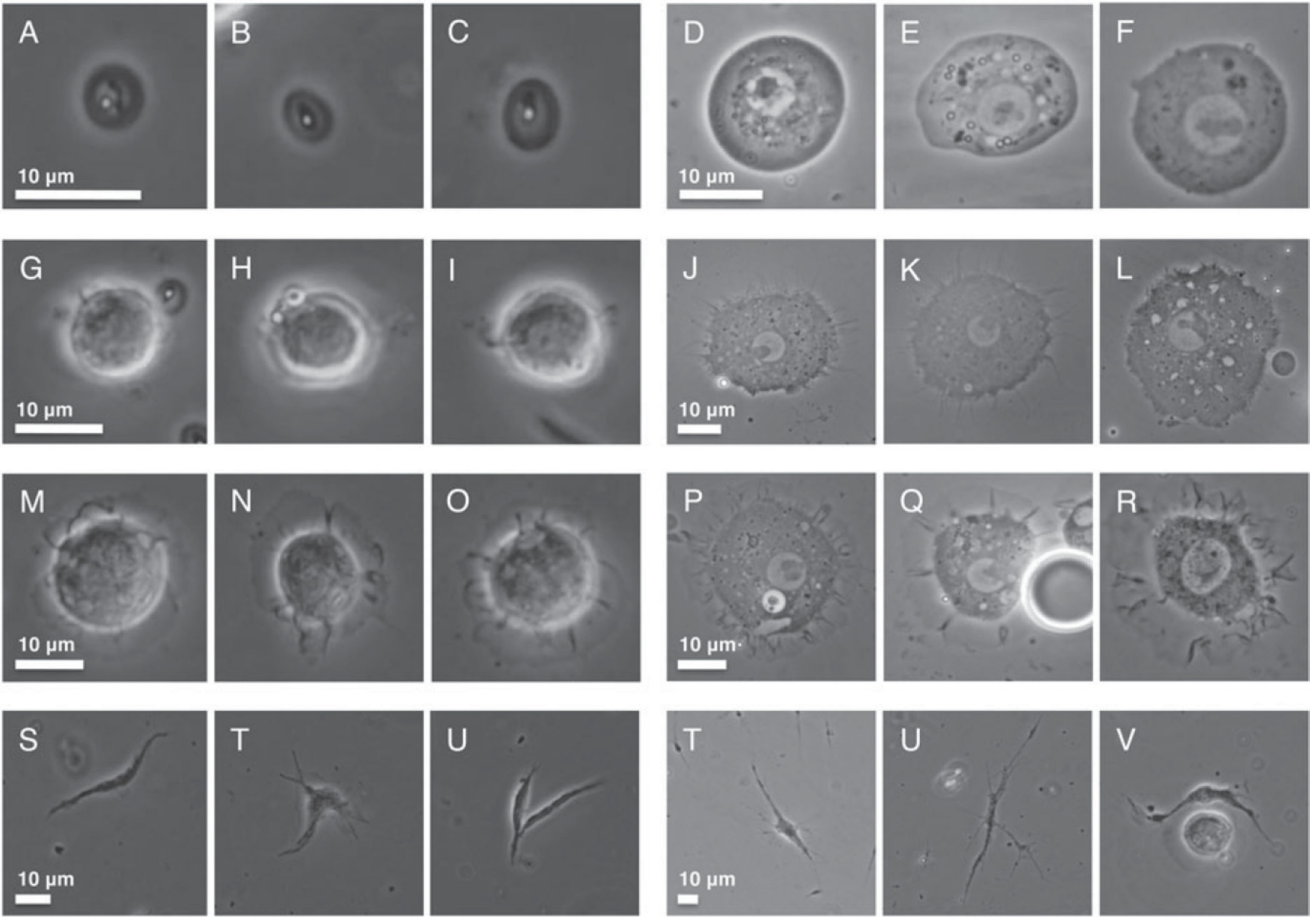
*Zaprionus indianus* haemocytes. Cells were dissected onto slides in 1× PBS buffer (A–C, G–I, M–O, S–U) or high density immersion oil (D–F, J–L, P–R, T–V). Six representative images are shown for plasmatocytes (A–F), podocytes (G–L), lamellocytes (M–R) and nematocytes (S–V). Size bars are consistent within treatments but note size bar variation across cell types and treatments.

**Fig. 10:**
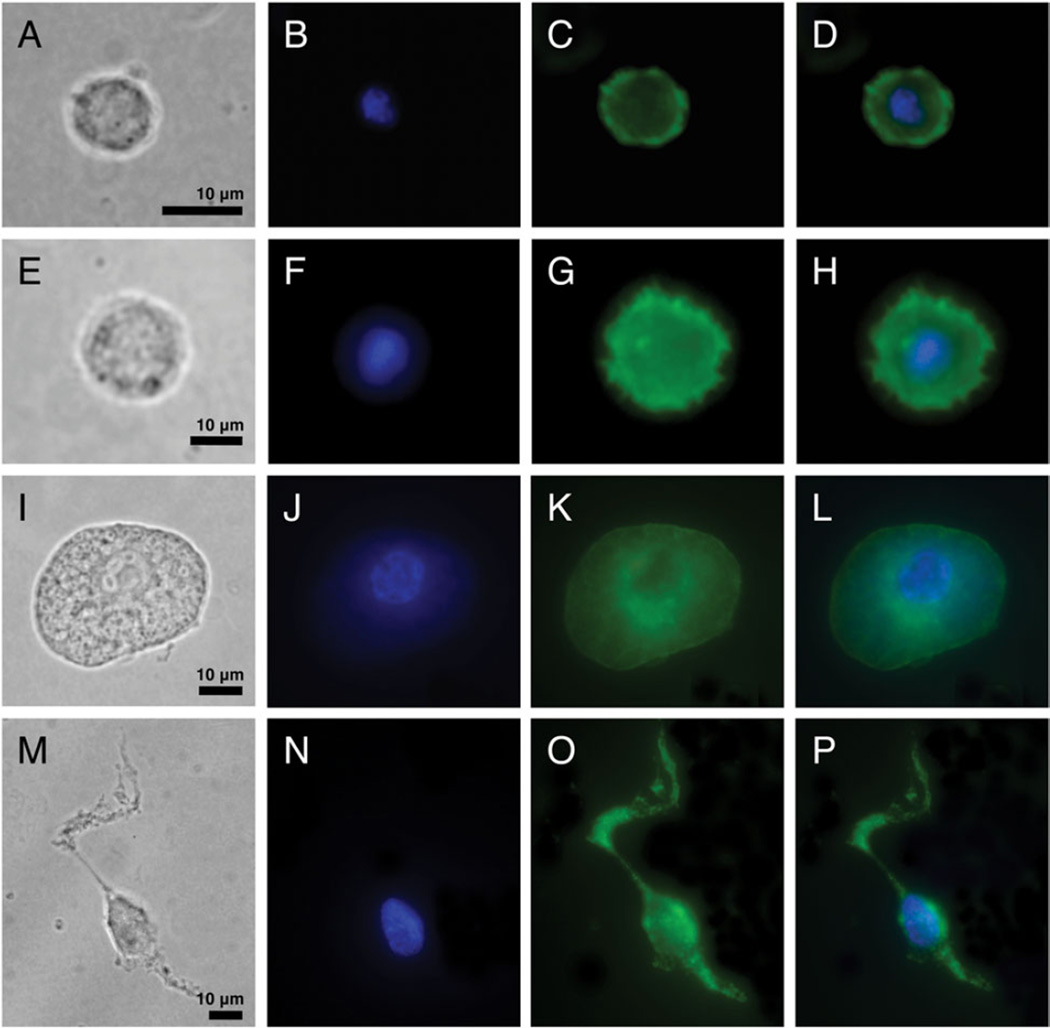
*Zaprionus indianus* haemocyte cytoskeletal and nuclear staining. A plasmatocyte (A–D), podocyte (E–H), lamellocyte (I–L), and nematocyte (M–P) are shown, in bright light (A, E, I, M), with DAPI nuclear staining (B, F, J, N), with TRITC-phalloidin actin staining (C, G, K, O), and with merged DAPI and TRITC-phalloidin stains (D, H, L, P). Note that haemocyte morphology differs somewhat from that shown in Fig. 9 due to the cell fixation process.

Because cellular encapsulation was found to be an important *Z. indianus* immune mechanism against the eggs of most parasitic wasp species, we characterized *Z. indianus* haemocytes in detail. Haemocytes were dissected from third instar *Z. indianus* larvae and visualized using two methods (Fig. 9). *Zaprionus indianus* has clear homologues of the plasmatocytes, podocytes and lamellocytes previously described from *D. melanogaster* (Rizki, 1957; Rizki and Rizki, 1980) (Fig. 9!A–R), but also has an extra haemocyte type termed nematocytes (Fig. 9!S–V), which are homologous to a previously described haemocyte type from *Drosophila willistoni* (Rizki, 1953) and army ants (Yeager, 1945). *Zaprionus indianus* plasmatocytes were classified as small round cells with an obvious nucleus and nucleolus, podocytes were classified as larger cells with numerous filopodia extending from the cell edge, lamellocytes were classified as even larger cells with a thin extended cytoplasmic region surrounding the main cell body, and nematocytes were classified as long spindly (fusiform) cells with variable numbers of long filopodia extending from the dominant cell axis. All images were taken from control larvae except those of lamellocytes, which were rarely found in uninfected larvae. Instead, lamellocyte images were taken from larvae pierced with a sterile needle, which mimics the cuticle wound associated with wasp infection (Markus *et al.* 2005). The cytoskeletal and nuclear structures of haemocytes were also visualized with TRITC-phalloidin and DAPI stains, respectively (Fig. 10). Like other haemocyte types, nematocytes were found to be mononuclear despite their large size, and also contained dense actin polymers to the full length of their long cytoplasmic projections.

**Fig. 11:**
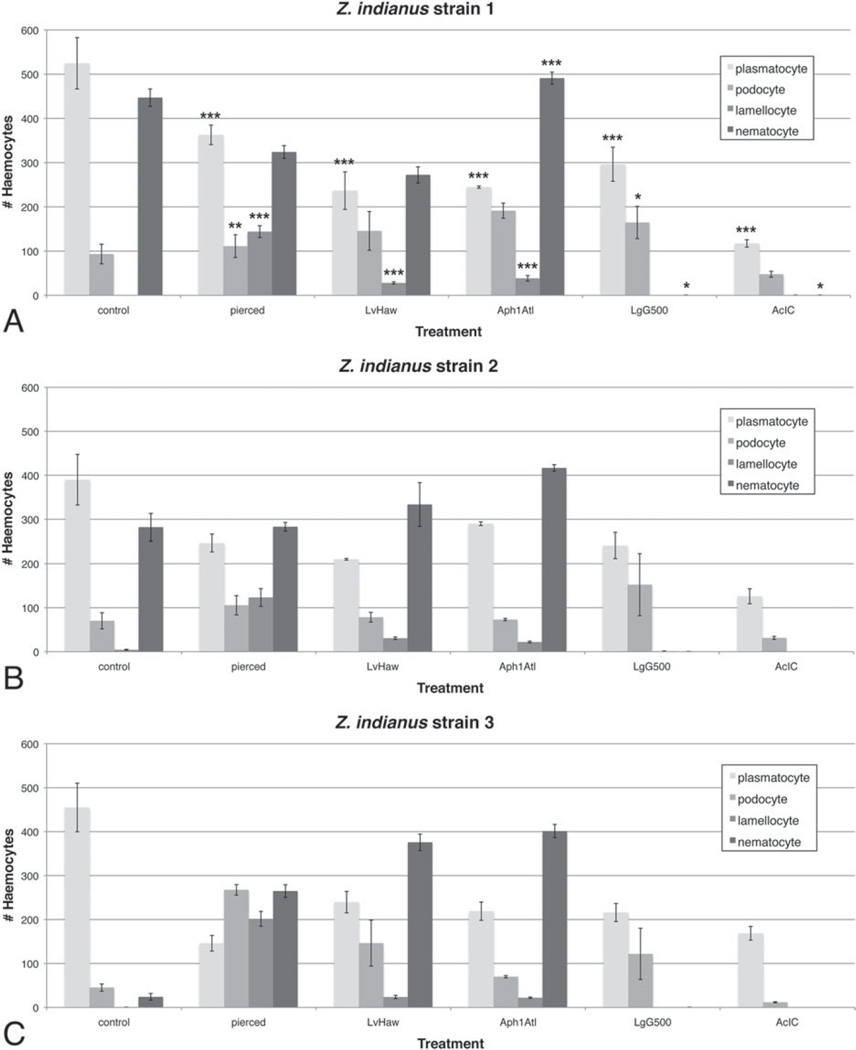
Constitutive and induced haemocyte numbers in *Z. indianus*. Control flies were treated like pierced flies but were not pierced; flies were also infected by four wasp strains that showed varying infection success in eclosion trials. The mean (±) standard error are shown for *Z. indianus* strains 1 (A), 2 (B), and 3 (C) based on five control and pierced replicates and three wasp-attack replicates. Significant differences between the control and each immune treatment for each cell type, using combined fly strain data, are shown in (A) with * <0·05, ** <0·01 and *** <0·001.

To assay potential associations between particular haemocyte types and response to wasp infection, haemocyte numbers were compared between control, pierced and wasp-infected *Z. indianus* larvae 24 h after challenge (Fig. 11). Four wasps were chosen: one that is regularly melanotically encapsulated by a successful fly immune response (*L. victoriae*), one that is regularly non-melanotically encapsulated by a successful fly immune response (*Aphaereta* sp.), and two that are rarely encapsulated and that have high infection success (*L. guineaensis* and *A. citri*). When haemocyte count data were combined across the three *Z. indianus* strains, we found significant decreases in plasmatocyte numbers following all immune treatments, which is likely explained by differentiation of plasmatocytes into podocytes and then into lamellocytes in response to wounding and wasp infection, as occurs in *D. melanogaster* (Rizki, 1957; Honti *et al.* 2009; Markus *et al.* 2009; Stofanko *et al.* 2010). Podocyte and lamellocyte numbers indeed significantly increased in pierced compared with control flies, although these effects were variable in wasp-infected flies, potentially due to their leaving circulation to form a capsule, or to immune suppression effects of wasp venoms. For example, we did not observe any circulating lamellocytes in flies infected by the virulent wasps *L. guineaensis* and *A. citri*, suggesting their venoms suppress lamellocyte differentiation and/or cause lamellocyte death. Thus, *L. guineaensis* and *A. citri*, which are from different Hymenopteran families, seem to have converged on similar immune suppressive infection strategies.

**Fig. 12:**
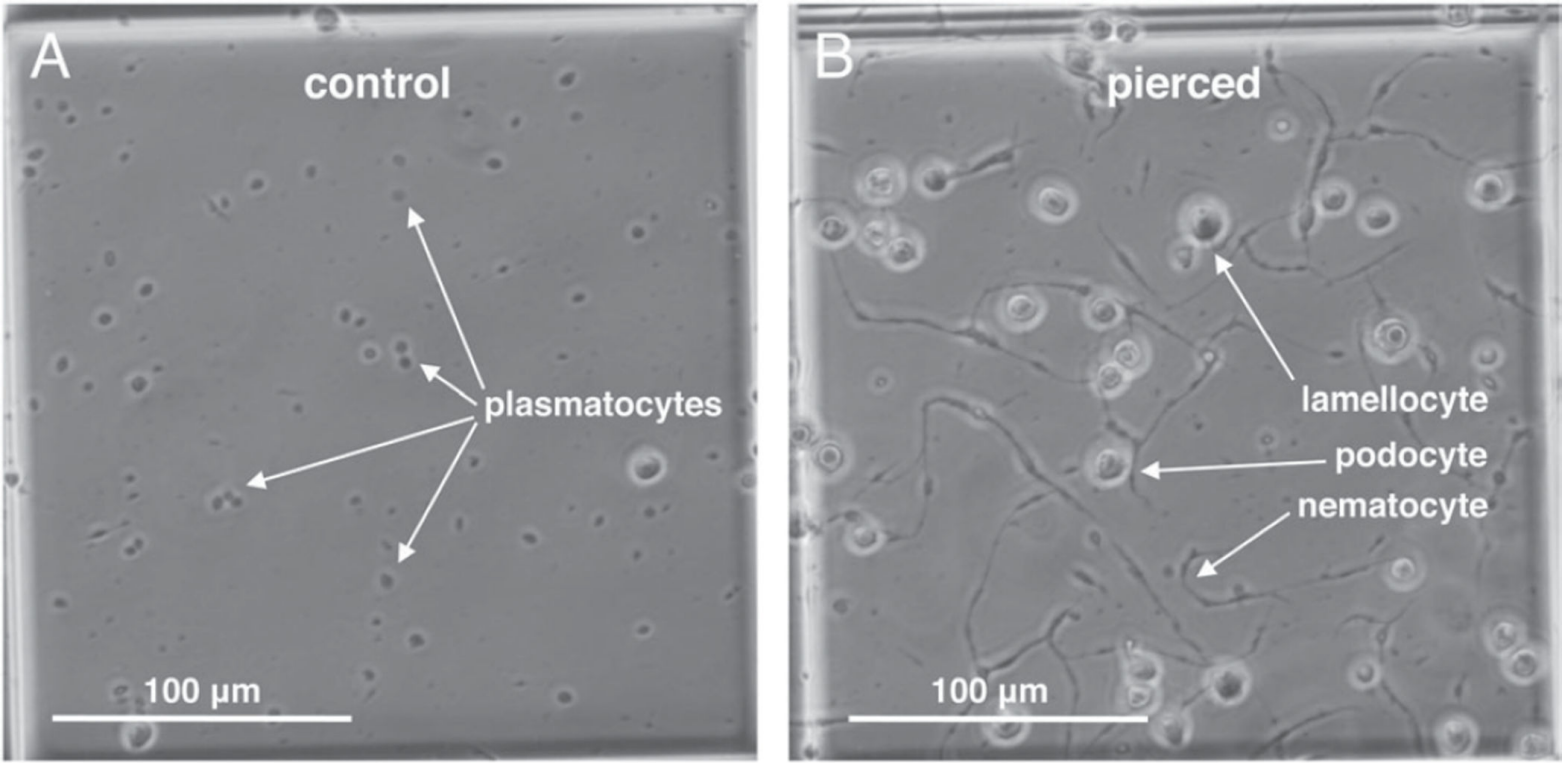
Induced production of nematocytes and other haemocyte types in *Z. indianus* strain 3. Haemocytes are visualized in a 0·25×0·25×0·1 mm haemocytometer field. In control flies (A) plasmatocytes are most numerous but 24 h after piercing (B) the numbers of podocytes, lamellocytes and nematocytes increase.

We found a significant increase in nematocyte numbers in *Z. indianus* larvae infected by the avirulent wasp *Aphaereta* sp. when nematocyte counts were combined across the three *Z. indianus* strains (Fig. 11), indicating nematocytes can be activated by wasp infection. There was also a significant loss of nematocytes in flies infected by the virulent wasps *L. guineaensis* and *A. citri* compared with controls, suggesting these wasps suppress nematocyte differentiation and/or cause nematocyte death as part of their virulence strategies. Surprisingly, *Z. indianus* strain 3 constitutively produced significantly fewer nematocytes than the other two strains, and then showed significant increases in nematocyte counts following piercing or infection by the avirulent wasps *L. victoriae* and *Aphaereta* sp. (all *P*<0·001) (Figs 11 and 12), providing further evidence that nematocyte production can be induced as part of the anti-wasp immune response. Altogether, these findings are a strong indicator that nematocytes are part of the anti-wasp immune response.

**Fig. 13:**
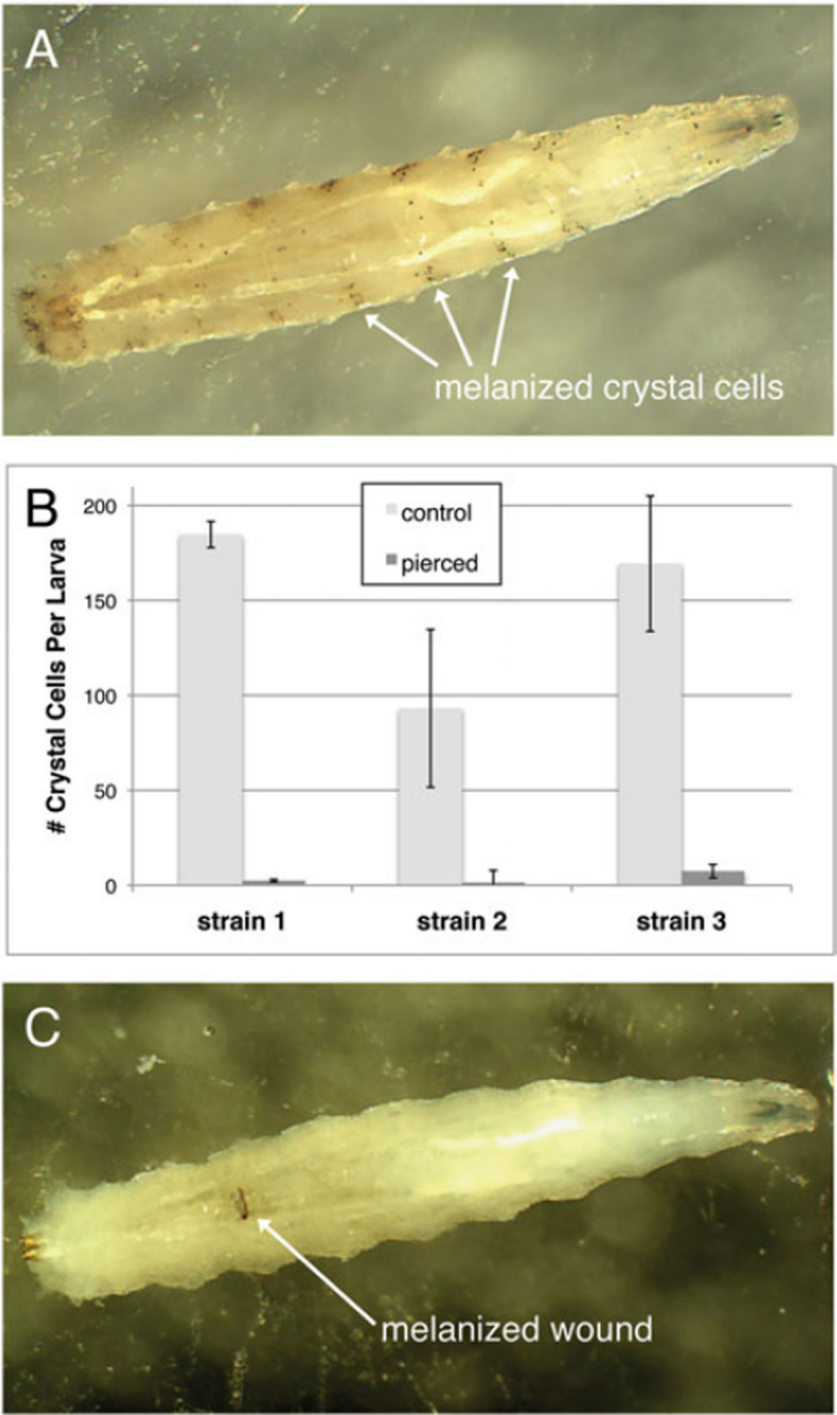
*Zaprionus indianus* crystal cells. Fly larvae were incubated to reveal melanized cells, presumably homologous to *D. melanogaster* crystal cells (A). The mean (±) standard error numbers of crystal cells were counted in control and pierced larvae from the three *Z. indianus* strains in three replicates each (B). Pierced, incubated fly larvae show melanized wounds and few melanized crystal cells (C).

Crystal cells are the fourth circulating haemocyte type found in *D. melanogaster* (in addition to plasmatocytes, podocytes, lamellocytes), and contain cytoplasmic crystals thought to be made up of the substrate for generating melanin. They can be difficult to image because they rapidly lose their defining characteristic, their crystals, when disturbed, secreting the crystal contents into the media. However, crystal cells can be counted easily because they self-melanize when fly larvae are incubated (Fig. 13A) (Rizki and Rizki, 1980; Williams *et al.* 2005). We counted crystal cells from control and pierced *Z. indianus* larvae, and found that strain 2 had significantly fewer constitutively produced crystal cells compared with the other two strains (Tukey contrasts, both *P*<0·001) (Fig. 13B). *Zaprionus indianus* showed a significant decrease in crystal cell counts following piercing (*P*<0·001), as in other Drosophila species (Kacsoh and Schlenke, 2012), likely due in part to the use of melanin in healing the pierce wound (Fig. 13C). Given wounding alone causes an absence of crystal cells, we did not count crystal cells from wasp-infected larvae.

### Nematocyte biology and species distribution

**Fig. 14:**
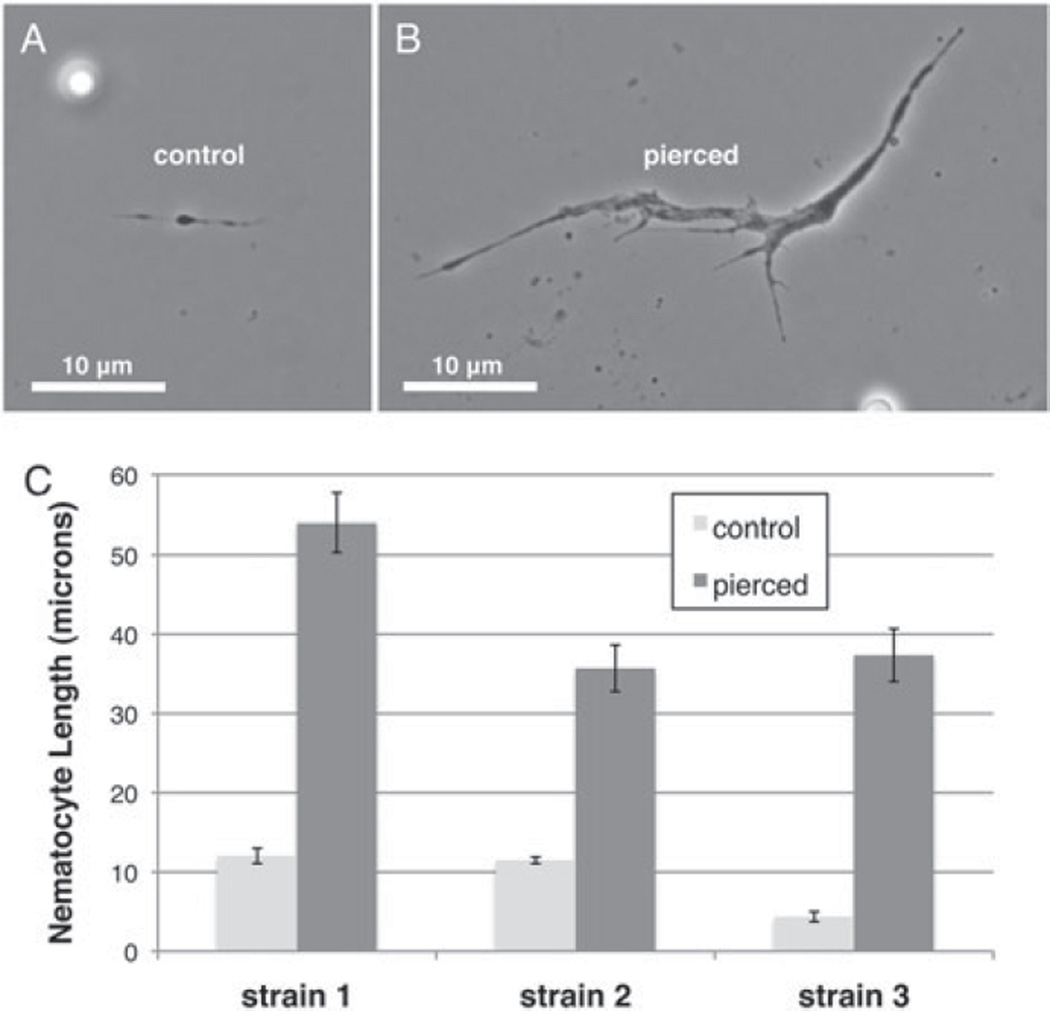
Constitutive and induced nematocyte length in *Z. indianus*. Nematocytes in un-induced flies (A) are much shorter and often show less branching than in wasp-attacked flies (B). The mean (±) standard error of nematocyte lengths from control and pierced flies (C) are based on three control and pierce replicates.

To follow up on the suggestion that nematocytes might play a role in the *Z. indianus* immune response against wasp eggs, we decided to further characterize these cells. We noticed that nematocytes appeared larger and grew more projections after fly larvae were challenged by piercing or with avirulent wasps. Thus, we compared nematocyte length in control and pierced flies (Fig. 14). We found a consistent significant increase in nematocyte length in pierced flies compared with control flies (ANOVA, *F* = 117·96, *P*<0·001) varying from 3- to 7-fold. This size change is likely not explained by a simple fusing of multiple nematocytes to one another, as staining revealed the presence of only a single nucleus in each activated nematocyte (Fig. 10).

**Fig. 15:**
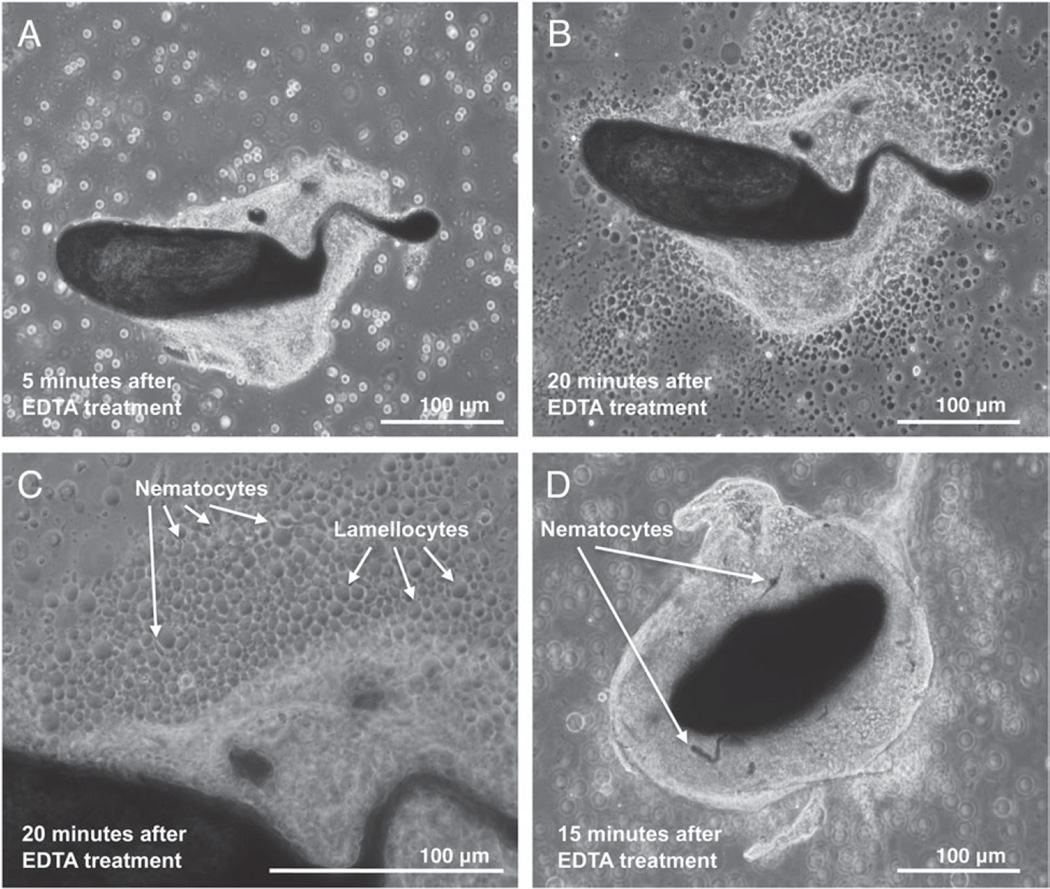
A role for nematocytes in encapsulation. Encapsulated wasp eggs dissected from fly hosts were treated with EDTA to disperse haemocytes. A single egg shown 5 min after treatment (A) and 20 min after treatment (B, C) has numerous lamellocytes and nematocytes making up the capsule. A second egg shown 15 min after EDTA treatment has clearly melanized nematocytes in the capsule (D).

It was previously shown that insect haemocytes involved in encapsulation of wasp eggs could be disassociated from the capsules and identified by incubating them in an EDTA buffer (Reik, 1968).We found this method also worked well for *Z. indianus* capsules (Fig. 15). Within 20 min, haemocytes from incubated capsules began to disperse, revealing that lamellocytes and nematocytes make up the majority of haemocyte types involved in encapsulation (Fig. 15C). Furthermore, we found that nematocytes involved in encapsulation were occasionally melanized (Fig. 15D), despite no cells of nematocyte shape being melanized in our crystal cell larval incubation experiment (Fig. 13).

**Fig. 16:**
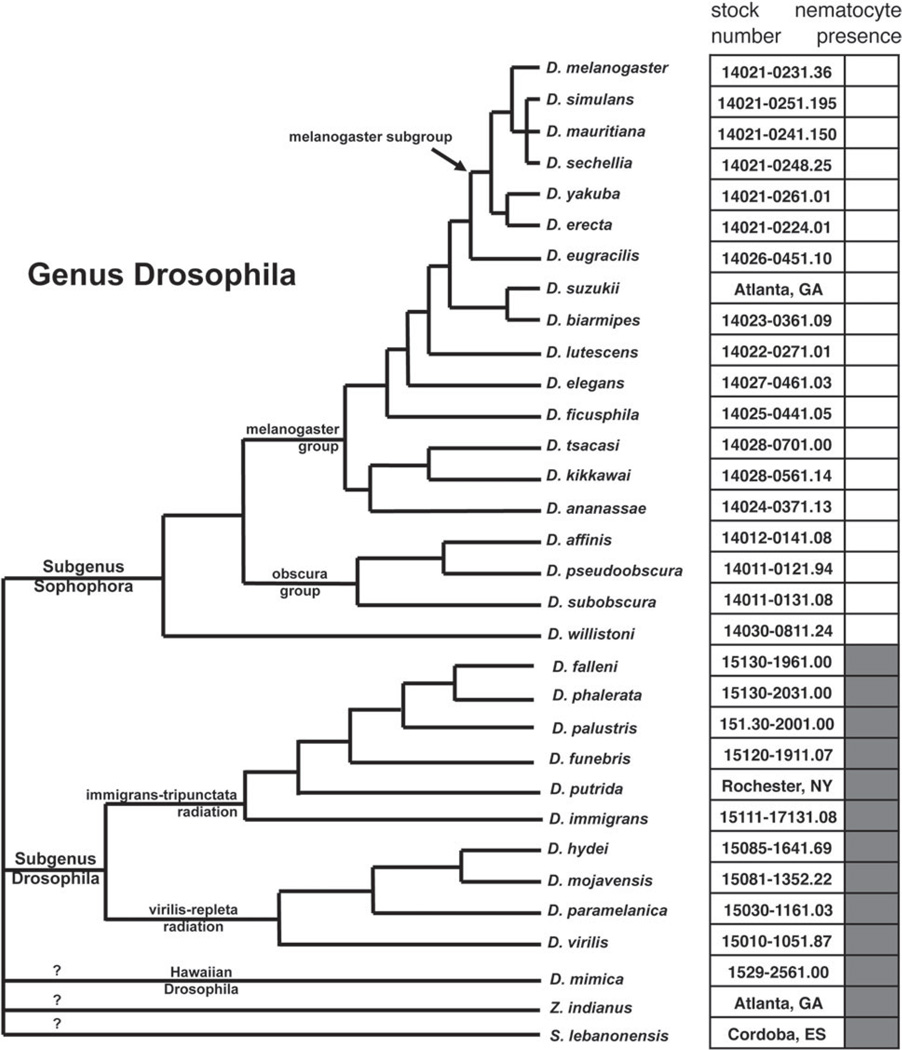
Presence of nematocytes across the Drosophila phylogeny. Dark boxes indicate fly species that produce nematocytes. Tree topology is a compilation based on numerous phylogenetic studies (Markow and O’Grady, 2006).

**Fig. 17:**
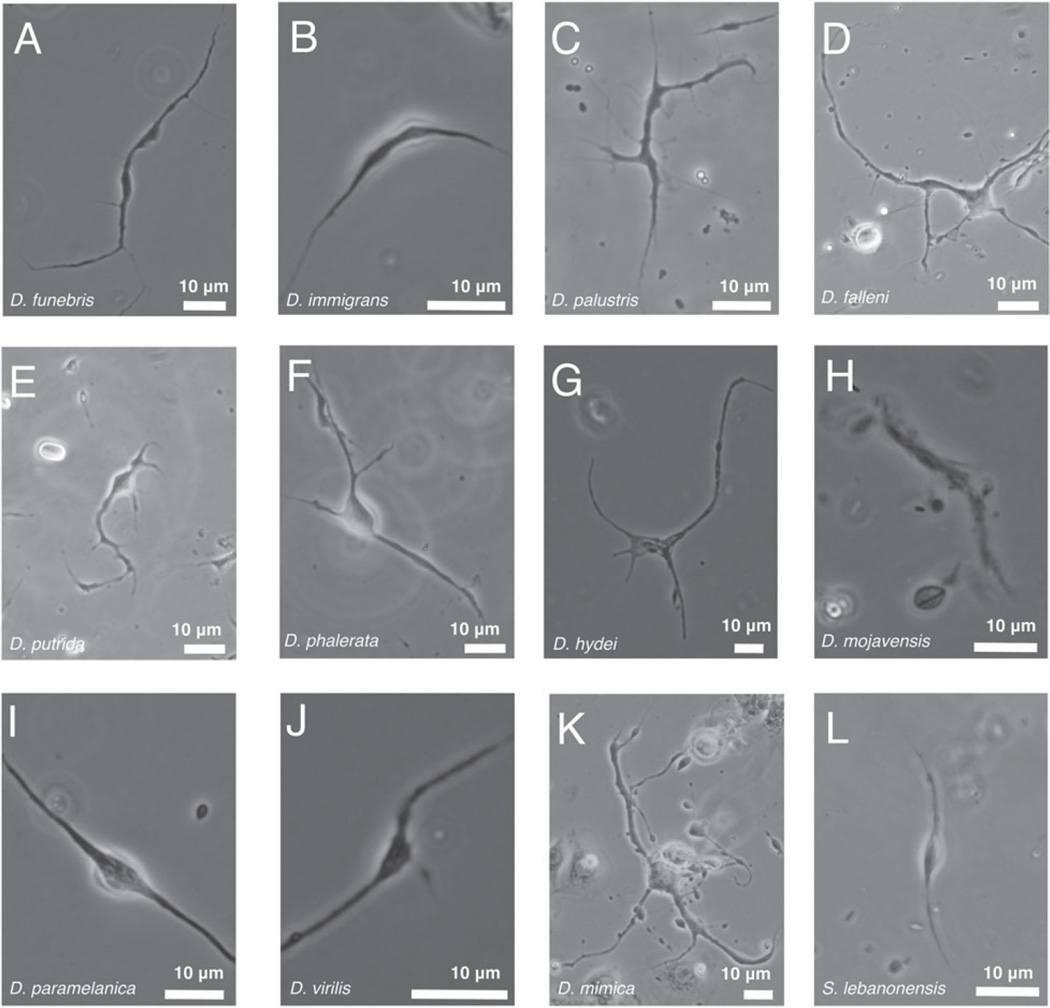
Nematocytes from other species in the subgenus Drosophila. Note size variation in constitutively produced nematocytes across species as indicated by variation in scale bar size.

The haemocyte composition of flies from the genus Drosophila, which includes the nominal genus Zaprionus, has been poorly characterized outside of the model organism *D. melanogaster. Drosophila melanogaster* does not produce a nematocyte-like haemocyte, and the only study that previously identified this cell type in a Drosophilid identified them from *D. willistoni* (Rizki, 1953). To better characterize the phylogenetic distribution of this unique cell type, we dissected haemocytes from a total of 27 Drosophilid species. We found nematocytes were constitutively produced in varying sizes by all members of the subgenus Drosophila that we tested, but were not found in any control or induced flies in the subgenus Sophophora, to which *D. melanogaster* belongs (Figs 16 and 17). Interestingly, we did not observe nematocytes in our strain of *D. willistoni*. From our data, it appears that nematocyte production is limited to a large clade within the genus Drosophila, but given the unknown position of the Zaprionus lineage within Drosophila we cannot yet determine whether nematocytes are a synapomorphy for the subgenus Drosophila.

## DISCUSSION

In previous work, Drosophila species from the melanogaster subgroup were found to have significantly different numbers of constitutively produced plasmatocytes, and there was a significant correlation (*r*^2^ = 0·90) between plasmatocyte counts and ability to melanotically encapsulate the eggs of the wasp *A. tabida* (Eslin and Prevost, 1998). Furthermore, we recently showed that *D. suzukii*, a member of the melanogaster group but not the melanogaster subgroup, produces far more haemocytes than *D. melanogaster* and is also significantly more resistant against a broad panel of parasitic wasps (Kacsoh and Schlenke, 2012). Haemocyte counts from *Z. indianus* (Fig. 11) are significantly higher than for *D. melanogaster* and only slightly lower than from *D. suzukii*. Correspondingly, *Z. indianus* has significantly greater success against our panel of parasitic wasps than *D. melanogaster*, but is not quite as resistant in general as *D. suzukii*. Altogether, this work suggests that high constitutive production of haemocytes is an effective and relatively simple mechanism by which hosts can evolve resistance to one of their most common groups of parasites.

Unlike other fly species for which the relationship between fly haemocyte count and resistance against parasitic wasps has been tested, *Z. indianus* can kill wasps by mechanisms other than melanotic encapsulation (Fig. 8). We found that *Z. indianus* encapsulates eggs of the wasp species *A. tabida* and *Aphaereta* sp. 1 by encapsulation without melanization. Non-melanotic encapsulation has been observed in other insect hosts (Salt, 1963, 1970) but not in the genus Drosophila: melanotic encapsulation appears to be the dominant anti-wasp immune response in the melanogaster group (Walker, 1959; Carton and Kitano, 1981; Kacsoh and Schlenke, 2012) and in individual species from other groups (Streams, 1968; Nappi, 1973, 1975a, b; Baker, 1979; Havard *et al.* 2009). Encapsulation appears to be absent or weak in many members of the obscura group (Baker, 1979; Eslin and Doury, 2006; Havard *et al.* 2009) and in individual species from other groups (Streams, 1968).

The melanica group, which is part of the virilis-repleta radiation in the subgenus Drosophila, appears to use humoral production of free radicals rather than encapsulation to kill wasp eggs (Streams, 1968; Nappi, 1970; Nappi and Streams, 1970; Carton *et al.* 2009). We also found that eggs of the wasp species *L. boulardi* can die in *Z. indianus* hosts in the absence of an obvious host cellular response, seemingly due to loss of their chorion layer. The *Drosophila paramelanica* killing mechanism was attributed to release of free radicals in the haemolymph around the wasp egg, but no observation of loss of wasp egg chorions was made (Carton *et al.* 2009). It can be very difficult to distinguish between a host humoral reaction responsible for killing the wasp egg or simply the unsuitability of a particular host species for successful wasp development (Salt, 1963). The variability we observed in the mechanisms by which *Z. indianus* killed wasp eggs suggests this fly may have redundant killing mechanisms, and that certain wasps are able to suppress certain subsets of these killing mechanisms. Alternatively, it is possible that *Z. indianus* tailors its immune response to the particular wasp it is infected by, despite the fact that *L. boulardi, A. tabida* and *Aphaereta* sp. 1 can all be melanotically encapsulated by other Drosophilids (Kacsoh and Schlenke, 2012). Either way, our data show there is specificity in fly–wasp interactions across fly hosts and wasp endoparasitoids.

Besides alternative wasp-killing mechanisms, *Z. indianus* also differs from *D. melanogaster* in its production of nematocytes. Several lines of evidence suggest *Z. indianus* uses nematocytes in its immune response against wasps, and in particular in encapsulation. First, nematocyte numbers are induced following immune challenge, particularly in *Z. indianus* strain 3 (Figs 11 and 12). Second, virulent wasps cause a complete loss of nematocytes in circulation, presumably because of venom-mediated mortality (Fig. 11). Third, nematocyte size greatly increases following an immune challenge, indicating nematocyte activation (Fig. 14). Fourth, nematocytes along with lamellocytes are actually present in the cellular capsules (Fig. 15). Altogether, our data begin to define the biological function of this poorly characterized class of Drosophila haemocyte.

Finally, *Z. indianus* has spread rapidly across North and South America and attained large population sizes in newly inhabited areas (Vilela, 1999; Goni *et al.* 2002; Tidon *et al.* 2003; Leao and Tidon, 2004; van der Linde *et al.* 2006). As a fig parasite, it has reduced fig production in many areas by 40–50%, and has decreased fig exports by up to 80% (Stein *et al.* 2003). Experimental studies testing the efficacy of various management strategies to contain the pest are lacking. One common biological control method is to introduce or supplement the natural enemies of the pest species. Parasitoid wasps have been used successfully to control numerous other arthropod pests (Huffaker, 1971; Debach, 1974; Clausen, 1978; Greathead, 1986; LaSalle and Gauld, 1993). In addition, up to 50% of Drosophila larvae collected in nature have been found to be infected by wasps, indicating they are one of the main selection pressures on juvenile flies (Janssen *et al.* 1988; Driessen *et al.* 1990; Fleury *et al.* 2004). The wasp species with the highest potential for use in biocontrol of *Z. indianus* that we identified (Fig. 4) were the larval endoparasitoids *L. guineaensis, Ganaspis* sp. 1, *Ganaspis* sp. 2 and *A. citri*, and the pupal endoparasitoid *Trichopria* sp. 1 (strain Tri1Fr in particular). However, much work would need to be done to understand any side-effects of these wasp species on members of the native fauna, as well as whether they would have the same high infection success in natural settings.
